# Acquired external auditory canal stenosis: clinical characteristics, surgical strategies and prognostic analysis

**DOI:** 10.3389/fped.2026.1814290

**Published:** 2026-05-14

**Authors:** CaiYun Meng, Ruosha Lai, WeiJing Wu, Shu Yang

**Affiliations:** Department of Otolaryngology, Head and Neck Surgery, The Second Xiangya Hospital of Central South University, Changsha, Hunan, China

**Keywords:** acquired external auditory canal stenosis, pediatric, restenosis, surgical strategy, traumatic

## Abstract

**Background:**

Acquired external auditory canal stenosis (EACS) is a major cause of conductive hearing loss in children and adults, with multiple etiologies including otological surgical procedures, blunt trauma, and chronic inflammatory diseases, which can lead to persistent hearing loss and other adverse outcomes. Due to children's unique anatomical fragility and strong tissue proliferative capacity, the clinical management of EACS remains challenging.This study aims to explore the clinical characteristics, optimal surgical approaches and prognostic factors of traumatic EACS in children, so as to provide evidence-based references for clinical practice.

**Methods:**

A retrospective medical record review was conducted for patients diagnosed with EACS who received treatment at The Second Xiangya Hospital between November 2020 and November 2025. Data were collected regarding age, gender, etiology, clinical symptoms, surgical methods, and postoperative outcomes. Descriptive statistics and Fisher's exact test were used for data analysis.

**Results:**

The findings indicated that the characteristics of EACS in children were unilateral involvement (100%), with the primary etiologies being associated with prior ear surgeries (86%) and isolated hearing loss (100%). The postoperative recurrence rate was 57%, and all recurrent cases were accompanied by restenosis. In pediatric surgeries, the temporoparietal fascial flap was the most frequently utilized graft (43%), 43% of patients underwent conchal cartilage resection, and 86% received absorbable drug—eluting stent (DES) implantation. All patients adhered to a unified DES implantation and postoperative care protocol. Type I tympanoplasty (Wullstein classification) was carried out in one pediatric case and one adult case for tympanic membrane repair. The adult cohort exhibited multiple etiologies (50% related to prior ear surgeries, 17% post—trauma, 25% post—inflammation), frequent accompanying symptoms (25% with tinnitus, 50% with otorrhea, 17% with earache), a recurrence rate of 25%, and no postoperative restenosis. Statistical analysis verified that the restenosis rate in pediatric patients was significantly higher than that in adults (*P* = 0.012), and there was no significant correlation between recurrence and flap selection, cartilage resection, or DES implantation (all *P* > 0.05).

**Conclusion:**

This study tentatively indicates that there exist disparities in the clinical characteristics of acquired external auditory canal stenosis between children and adults. The risk of postoperative restenosis in children is notably elevated, and this tendency might be associated with the inherent anatomical fragility and robust tissue proliferation capacity in children. Comprehensive preoperative imaging assessment, individualized surgical plan development, standardized drug—eluting stent implantation, structured long—term postoperative follow-up, and postoperative care may be conducive to enhancing the prognosis of acquired external auditory canal stenosis in children. Multicenter prospective studies with a larger sample size are required to further validate the optimal treatment strategy for acquired external auditory canal stenosis in children.

## 1 Introduction

Acquired aural stenosis, an unusual etiology of conductive hearing loss, was categorized into four distinct subtypes by Tos et al.: post-traumatic, post-surgical, neoplastic, and post-inflammatory. This classification system is widely referenced in otological literature for its clinical relevance to etiological diagnosis and therapeutic decision-making (1). Compared with congenital aural stenosis, acquired aural stenosis, though rare, typically develops subsequent to motor vehicle collisions, gunshot wounds, radiation therapy, or otological surgeries (2–4). Its pathological process is a fibroproliferative inflammation progressing over several years,it initiating with the loss of the squamous epithelial layer of the tympanic membrane and the medial auditory canal, subsequently triggering inflammation and granulation tissue formation (5, 6). The ultimate outcome is the circumferential loss of the epidermis lining the auditory canal, leading to granulation tissue deposition, fibrosis, and stenosis (5). Clinical manifestations typically include otorrhea and hearing loss, with surgical intervention being the standard of care for these conditions. The primary goals of surgery are to prevent complications and restore auditory function. While several surgical techniques have been described in the literature, EAC canaloplasty remain the mainstay approaches for achieving external auditory canal (EAC) widening (7–9). Nevertheless, there are limited studies on acquired external auditory canal stenosis in the pediatric population.

This study through an exploration of core indicators, including clinical manifestations, disease course, surgical strategies, and postoperative management, the objective was to examine the correlation between EACS and its etiologies. This study intended to highlight the significance of etiology management and prevention in clinical practice to attain favorable clinical outcomes.

## 2 Materials and methods

### 2.1 Study population and control group

A retrospective review was conducted on the clinical records of patients diagnosed with acquired EACS at The Second Xiangya Hospital of Central South University from November 2020 to November 2025. Inclusion criteria: (1) Confirmed acquired external auditory canal stenosis and atresia (EACS/EACA) diagnosis via EAC endoscopy and temporal bone computed tomography (CT); (2) Complete clinical, surgical, and follow-up data; (3) Pediatric patients aged ≤18 years, adult patients aged >18 years. Exclusion criteria: (1) Congenital EACS; (2) Neoplastic-induced EACS; (3) Incomplete clinical data.

A total of 19 patients were enrolled, including a study group of seven pediatric patients with EACS and a control group of 12 adult patients with EACS treated during the same period.

This study was approved by the Ethics Committee of The Second Xiangya Hospital of Central South University, and informed consent was obtained from all patients or their legal guardians for the use of clinical data for research purposes. All clinical data were de-identified to protect patient privacy.

### 2.2 Clinical evaluation and diagnosis

All patients received a comprehensive otological evaluation, including medical history review, physical examination, external auditory canal endoscopy, and temporal bone computed tomography (CT) scanning.

The diagnostic criteria for acquired external auditory canal stenosis applied in this study were as follows: (1) Endoscopy showed stenosis (stenotic type) or complete occlusion (atresia type) of the external auditory canal lumen; (2) CT demonstrated fibrosis, bone hyperplasia, or soft tissue obstruction in the external auditory canal, with no evidence of congenital malformations.

Recurrence was defined as the reappearance of acquired external auditory canal stenosis after initial surgical treatment, meeting at least one of the following criteria: (1) Recurrence or worsening of hearing loss; (2) New onset or recurrence of otorrhea, tinnitus, or earache; (3) Postoperative re-stenosis that required further intervention.

Re-stenosis was diagnosed when endoscopy revealed stenosis of the external auditory canal lumen and CT confirmed fibrous proliferation or bony obstruction.

Postoperative follow-up was performed at the otology outpatient clinic. Follow-up was scheduled at 1 week, 1 month, 3 months, 6 months after surgery, and annually thereafter. Some patients were lost to follow-up (e.g., due to relocation or poor compliance), resulting in incomplete long-term follow-up data (>12 months), which might have affected the assessment of long-term postoperative outcomes.

### 2.3 Surgical reconstruction techniques

All surgical procedures were carried out by senior otolaryngologists who possess expertise in ear surgeries. The primary objectives are to reconstruct the patency of the external auditory canal, restore auditory function, and prevent restenosis. According to the surgical records of all enrolled patients, no patient underwent auditory reconstruction surgery, the clinical management for hearing loss in this cohort was focused on resolving the mechanical obstruction of the external auditory canal via canaloplasty and related procedures, which is the fundamental measure to alleviate conductive hearing loss caused by EACS. The detailed surgical reconstruction techniques are as follows:

#### 2.3.1 Basic surgical approaches

Auricular atresia canalplasty is the definitive surgical intervention for aural atresia, aimed at establishing a functional, epithelialized EAC. The procedure comprises four key components: (1) complete resection of the atresia plate—including both bony and cartilaginous elements; (2) precise excision of the medial EAC skin and the squamous epithelial layer of the tympanic membrane, with deliberate preservation of the fibrous lamina to maintain structural competence; (3) controlled bony enlargement of the EAC lumen, performed to maximize diameter while strictly avoiding violation of the mastoid cortex or exposure of mastoid air cells; and (4) mucosal reconstruction via autologous split-thickness skin grafting, followed by meticulous, layered packing of the EAC with antibiotic-impregnated, non-adherent material to support re-epithelialization and prevent restenosis (10). Type I tympanoplasty is indicated for patients with uncomplicated, non-retracted tympanic membrane perforations classified under the Wullstein system (11)—a validated, anatomically grounded classification widely employed in otologic literature and clinical practice to guide graft selection, surgical planning, and outcome prediction. This technique utilizes autografts—most commonly temporalis fascia or tragal perichondrium—to restore tympanic membrane continuity and reconstitute the acoustic impedance-matching function of the middle ear.Modified radical mastoidectomy (MRM) is reserved for cases with confirmed chronic mastoiditis or cholesteatoma-related mastoid air cell disease; it achieves comprehensive removal of diseased mucosa, granulation tissue, and epidermal matrix, restores patency of the aditus ad antrum and attic, and establishes durable middle ear ventilation (12).

#### 2.3.2 Flap transplantation for mucosal reconstruction

The temporalis fascial flap is the preferred graft for EAC mucosal reconstruction. Standardized technique includes: (1) a curved incision 2 cm above the zygomatic arch; (2) subperiosteal harvest of fascia with preservation of the superficial temporal artery; (3) intraoperative sizing and transposition to match the defect's extent and shape; and (4) secure fixation with absorbable monofilament sutures to achieve a watertight, tension-free, epithelialized lining. Alternative grafts—conchal bowl, scalp, or Thiersch—are used second-line when anatomy, defect features, or patient factors contraindicate the temporalis flap; harvesting is adapted to each donor site's vascular and structural constraints. The Thiersch graft is a thin, full-thickness free skin graft from the inner thigh or postauricular region (10), sized to the EAC defect. In children, grafts measure 0.5–1.0 cm wide × 1–3 cm long and are trimmed intraoperatively to fit the narrow, curved pediatric EAC. Grafts are oversized by 10%–15% to offset tissue recoil and contraction. After harvest, grafts are carefully excised from subcutaneous fat using ophthalmic scissors under direct vision, then immediately placed in sterile saline to preserve viability.

#### 2.3.3 Cartilage resection

In patients with severe EAC stenosis, targeted resection of conchal cartilage or tragal cartilage serves as an adjunctive surgical strategy to augment canal lumen diameter. The technique is performed via a transcanal or endaural approach: (1) precise exposure of the conchal cartilage through a minimally invasive endaural incision; (2) controlled excision of a defined cartilaginous segment—using either fine bone-cutting forceps or sterile ophthalmic scissors—under direct visualization to achieve calibrated enlargement of the EAC without compromising structural support; and (3) meticulous approximation of the residual cartilage edges with absorbable sutures to minimize postoperative hematoma formation, prevent fibrous adhesion, and preserve both auricular contour and biomechanical integrity.

#### 2.3.4 Drug-eluting stent (DES) implantation

A standardized, protocol-driven drug-eluting stent (DES) implantation strategy was implemented for all enrolled patients. The procedure comprises the following rigorously defined steps: (1) Selection of an absorbable DES (Shanghai Pu Yi Co., Ltd.; model SDES2514-1; *in vivo* degradation time: 30 days) tailored to the individual patient's EAC dimensions—specifically its luminal diameter and axial length; (2) Controlled loading of the DES with a sustained-release corticosteroid formulation (652 µg total dose), selected for its potent anti-proliferative and anti-fibrotic activity to mitigate postoperative granulation tissue formation, fibroblast hyperplasia, and pathological cicatrization; (3) Precise endoscopic placement of the DES within the mid-EAC segment—defined anatomically as the region extending from the isthmus to the medial two-thirds of the bony canal—to ensure optimal mechanical support and localized drug delivery; and (4) Scheduled stent exchange at monthly intervals: degraded remnants are meticulously debrided under microscopic visualization, and a fresh DES is implanted; clinical and endoscopic surveillance continues for 3–6 months until complete, confluent epithelialization of the EAC mucosa is confirmed.

#### 2.3.5 Packing material placement

Immediately following DES implantation, the EAC is packed with iodoform-impregnated gauze—extended from the meatal orifice to the tympanic membrane—to provide adjunctive mechanical stabilization, prevent graft or flap displacement, and inhibit postoperative synechiae formation. The gauze is uniformly impregnated with a broad-spectrum antibiotic ointment to minimize epithelial shear stress and confer localized antimicrobial prophylaxis. Scheduled replacement is performed biweekly during the first postoperative month under otomicroscopic guidance to maintain optimal moisture balance, ensure unobstructed drainage, and facilitate progressive re-epithelialization of the EAC mucosa (13).

### 2.4 Statistical analysis

All statistical analyses were conducted in IBM SPSS Statistics v26.0. Baseline and outcome data are summarized as frequencies (%), means ± SD, or medians (IQR). Categorical variables were compared using Fisher's exact test (2 × 2) or Fisher–Freeman–Halton test (>2 × 2). Partial eta-squared (*η*^2^) from univariate ANOVA quantified the association between cartilage resection and recurrence; *η*^2^ ≥ 0.01/0.06/0.14 indicated small/medium/large effects. Statistical significance was set at two-sided *p* < 0.05. Given the small sample—especially in the pediatric group—findings were interpreted conservatively to account for reduced power and elevated type II error risk. Multivariable modeling was omitted due to insufficient events-per-variable ratio (<10:1).

## 3 Result

### 3.1 General clinical characteristics

A total of 19 patients diagnosed with acquired EAC stenosis were enrolled. All subjects had complete baseline clinical documentation and ≥ six months of structured postoperative follow-up, with no missing data for primary endpoints. Tables 1–5 summarize the baseline demographic characteristics, etiologies, clinical features, and follow-up data of the pediatric cohort (*n* = 7) and adult cohort (*n* = 12). Notably, no cases of congenital EAC atresia or stenosis were identified; all included patients exhibited acquired pathology attributable to one of three mutually exclusive etiologic categories: (i) iatrogenic—secondary to prior otologic surgery; (ii) post-traumatic—following blunt or penetrating trauma; or (iii) post-inflammatory—arising from chronic otitis externa or granulomatous disease. Detailed surgical histories are provided in Supplementary Table S1.

**Table 1:** Baseline clinical characteristics of individual pediatric patients with acquired external auditory canal stenosis.

Case no	Sex	Age (years)	Side	Symptom	Etiology	Treatment
1	F	8	R	HL	Post-otologic surgery	Canaloplasty
2	F	14	L	HL	Post-otologic surgery	Canaloplasty
3	M	17	L	HL	Post-otologic surgery	Type I Tympanoplasty
4	M	14	R	HL	Post-otologicsurgery	Canaloplasty,MRM
5	M	8	R	HL	Post-otologic surgery	Canaloplasty
6	M	8	L	HL	Post-otologic surgery	Canaloplasty
7	M	7	L	HL	RTA	Canaloplasty

M, male; F, female; R, right; L, left; RTA, Blunt trauma due to road traffic accident; MRM, Modified Radical Mastoidectomy; HL, Hearing Loss.

**Table 2:** Clinical characteristics of adult patients with post-traumatic and post-inflammatory acquired external auditory canal stenosis.

Case no	Sex	Age (years)	Side	Symptom	Etiology	Treatment
1	M	55	L	HL、Tinnitus	PI-EACS	Canaloplasty、Type I Tympanoplasty
2	F	54	B	HL、Otorrhea	PI-EACS	Canaloplasty
3	M	65	R	HL、Otorrhea	PTS	Canaloplasty
4	F	53	R	HL、Otorrhea	Post-otologic surgery	
5	F	51	L	HL、Tinnitus	Post-otologic surgery	Canaloplasty
6	M	44	L	HL、Otorrhea	PI-EACS	Canaloplasty、MRM
7	F	45	R	HL	PI-EACS	Canaloplasty
8	F	41	L	HL、Otorrhea	Post-otologic surgery	Canaloplasty
9	M	31	R	HL、Otalgia	Post-otologic surgery	Canaloplasty
10	F	69	R	HL、Otalgia	PTS	Canaloplasty
11	F	46	L	HL、Otorrhea	Post-otologic surgery	Canaloplasty
12	F	60	L	HL、Tinnitus	Post-otologic surgery	Canaloplasty

M, male; F, female; R, right; L, left; B, Bilateral; RTA, Blunt trauma due to road traffic accident; MRM, Modified Radical Mastoidectomy; HL, Hearing Loss; PI-EACS, Post-Inflammatory External Auditory Canal Stenosis; PTS, Post-Traumatic Stenosis.

**Table 3:** Comparison of clinical data between pediatric and adult patients with EACS.

Characteristic	Pediatric (*n* = 7)	Adult (*n* = 12)
Sex		
Male	5 (71%)	4 (33%)
Female	2 (29%)	8 (67%)
Recurrence rate (%)	4 (57%)	3 (25%)
Involvement side		
Right	3 (43%)	5 (42%)
Left	4 (57%)	6 (50%)
Bilateral	0	1 (8%)
Etiology		
Post-otologic surgery	6 (86%)	6 (50%)
Post-traumatic	1 (14%)	2 (17%)
Symptoms		
Hearing loss	7 (100%)	12 (100%)
Tinnitus	0	3 (25%)
Otorrhea	0	6 (50%)
Otalgia	0	2 (17%)

**Table 4:** Comparative analysis of surgical methods for EACS in adults and children.

Variable	Pediatric (*n* = 7)	Pediatric recurrence group (*n* = 4)	Adults (*n* = 11, 1 cases not operated excluded)	Adult recurrence group (*n* = 3)	*P* Value = 0.05
Selection of flap					
Temporalis fascial	3 (43%)	1 (25%)	1 (9%)	0	*P* = 0.616, *P* > 0.05
Auricular fossa	1 (14%)	1 (25%)	1 (9%)	0	
Scalp flap	1 (14%)	1 (25%)	1 (9%)	0	*P* = 0.326 > 0.05
Postauricular thin-layer free	1 (14%)	1 (25%)	0	0	
EAC posterior wall canal	1 (14%)	0	6 (55%)	2 (67%)	
Stent implantation	6 (86%)	3 (75%)	6 (55%)	1 (33%)	
Cartilage resection					
Auricular fossa	3 (43%)	3 (75%)	3 (27%)	1 (33%)	*P* = 0.594, P > 0.05
Tragal	1 (14%)	1(25%)	0	0	

**Table 5:** Comparison of postoperative efficacy between adults and children.

	Pediatric (*n* = 7)				
Parameter	Pediatric (*n* = 7)	Pediatric recurrence group (*n* = 4)	Adults (*n* = 11,1 case not operated excluded)	Adult recurrence group (*n* = 3)	Statistical values (pediatrics vs. adults) ***α*** = 0.05
Sex					*P* = 0.198 > 0.05
Male	5	2 (50%)	4 (33%)	1 (33%)	*P* = 0.481 > 0.05
Female	2	2 (50%)	8 (67%)	2 (67%)	
Otorrhea	0	0	2 (18%)	2 (67%)	
Postoperative restenosis	4	4 (100%)	0	0	*P* = 0.012 < 0.05

Among the six pediatric patients with iatrogenic stenosis, MRM was the predominant antecedent procedure (4/6, 67%), followed by primary EAC canalplasty (1/6, 17%) and posterior auricular soft-tissue tumor excision (1/6, 17%). The latency period between initial surgery and stenosis manifestation ranged from 6 months to 12 years (median: 5.0 years). Audiometric evaluation confirmed unilateral conductive hearing loss in all pediatric patients; none reported tinnitus, otorrhea, or otalgia at presentation. Recurrence—defined as radiologically or endoscopically confirmed re-stenosis requiring reintervention—occurred in four of seven pediatric patients (57.1%), exclusively among those with iatrogenic etiology; all recurrences were characterized by progressive luminal narrowing without epithelialization failure.

The adult cohort (*n* = 12) had a mean age of 51.2 ± 14.8 years, with a female predominance (8/12, 67%). Laterality distribution was right-sided in five patients (42%), left-sided in six (50.0%), and bilateral in one (8%; left stenosis with contralateral complete atresia). Etiology was heterogeneous: iatrogenic (6/12, 50%), post-traumatic stenosis (PTS; 2/12, 17%), and post-inflammatory acquired EAC stenosis (PI-EACS; 4/12, 33%). As detailed in Supplementary Table S1, MRM accounted for four of six iatrogenic adult cases (67%), while posterior auricular tumor excision comprised the remainder (2/6, 33%). The median latency from index surgery to stenosis onset was significantly shorter than in the pediatric group (2.0 vs. 5.0 years; range: 0.5–12 years). All adult patients presented with conductive hearing loss; comorbid symptoms included tinnitus (3/12, 25%), otorrhea (6/12, 50%), and otalgia (2/12, 17%). Recurrence occurred in 3 of 12 adults (25%), all following iatrogenic etiology; however, unlike the pediatric cohort, none developed anatomical re-stenosis—recurrent symptoms were attributed to persistent inflammation or granulation tissue without measurable lumen reduction.

### 3.2 Surgical reconstruction techniques: study vs. control group

Data from seven pediatric and 11 adult patients were analyzed; one adult declined surgery and was excluded. Surgical techniques and implant use are detailed in Table 4. Percentages are reported as whole numbers. Both groups were received identical drug-eluting stent implantation and postoperative care—including scheduled stent exchanges, topical antimicrobial prophylaxis, and otomicroscopic surveillance—over a fixed three–six month period. Care duration, procedures, and protocol adherence were equivalent between groups.

#### 3.2.1 Flap selection

Children's group: The temporalis fascial flap was the most frequently utilized graft (3/7, 43%), followed by the auricular fossa, scalp, postauricular thin-layer free, and EAC posterior wall flaps—each employed once (1/7, 14%). In the pediatric recurrence subgroup (*n* = 4), all four flaps were represented equally (1 case each, 25%), whereas the EAC posterior wall flap was not used.

Adult group: The EAC posterior wall flap was the predominant graft (6/11, 55%), while the temporalis fascial, auricular fossa, and scalp flaps were each used once (1/11, 9%); the postauricular thin-layer free flap was not employed. Among the three adults with recurrence, two (67%) received the EAC posterior wall flap.

Fisher's exact test revealed no statistically significant association between flap type and postoperative recurrence (*P* = 0.616).

#### 3.2.2 Cartilage resection

Children's group: Cartilage resection was performed in 4 of 7 patients (57%), including auricular fossa resection in 3 (43%) and tragal resection in 1 (14%). Among the 4 pediatric patients with recurrence, 3 (75%) underwent auricular fossa resection and 1 (25%) underwent tragal resection.

Adult group: Cartilage resection was performed in 3 of 11 patients (27%), all involving the auricular fossa; no adult underwent tragal resection. Notably, none of the three adults with recurrence received cartilage resection.

Fisher's exact test indicated no statistically significant association between cartilage resection and recurrence (*P* = 0.594). Partial eta-squared (*η*² = 0.089; 95% CI: 0.000–0.368) further confirmed a negligible clinical effect of cartilage resection on recurrence risk.

#### 3.2.3 Drug-eluting stent (DES) implantation

Children's group: Stent implantation was performed in 6 of 7 patients (86%), all receiving absorbable, steroid-eluting drug-eluting stents. Among the 4 pediatric patients with recurrence, three (75%) had stents implanted.

Adult group: Stent implantation was performed in 6 of 11 patients (55%). Identical stent specifications (diameter, length, degradation profile) and standardized retention duration (3–6 months) were maintained across both age groups. Among the 3 adults with recurrence, 1 (33%) received a stent.

Fisher's exact test revealed no statistically significant association between drug-eluting stent implantation and postoperative recurrence (*P* = 0.326).

## 4 Discussion

To our knowledge, this is among the few studies to comprehensively characterize the clinical features, surgical approaches, and prognostic determinants of acquired EACS in pediatric patients—and to directly compare these with an adult cohort. This retrospective analysis of seven pediatric and 12 adult patients aimed to inform evidence-based, age-stratified management strategies for EACS.

Acquired EACS exhibited distinct epidemiological patterns across age groups, closely tied to etiological differences. In our cohort, the pediatric group demonstrated a male predominance (71%) and a mean age of 11 years. Notably, 86% of pediatric cases were attributable to prior otologic surgery—significantly exceeding the 50% observed in adults (*P* < 0.05). This aligns with established pathophysiological principles: the pediatric EAC mucosa is inherently more delicate, susceptible to iatrogenic injury during surgery, and exhibits heightened proliferative capacity—factors that collectively predispose to postoperative stenosis (14). In contrast, adult etiologies were heterogeneous: prior otologic surgery (50%), post-traumatic (17%), and post-inflammatory (25%). All pediatric cases were unilateral; bilateral involvement occurred in 8% of adults—a pattern likely reflecting cumulative multifactorial insults over time. Critically, all enrolled cases represented acquired (not congenital) EACS, rigorously excluding developmental anomalies. Importantly, surgery-related EACS was recognized as an expected, non-preventable consequence of normal tissue repair—not a marker of technical error.

**Figure 1:**
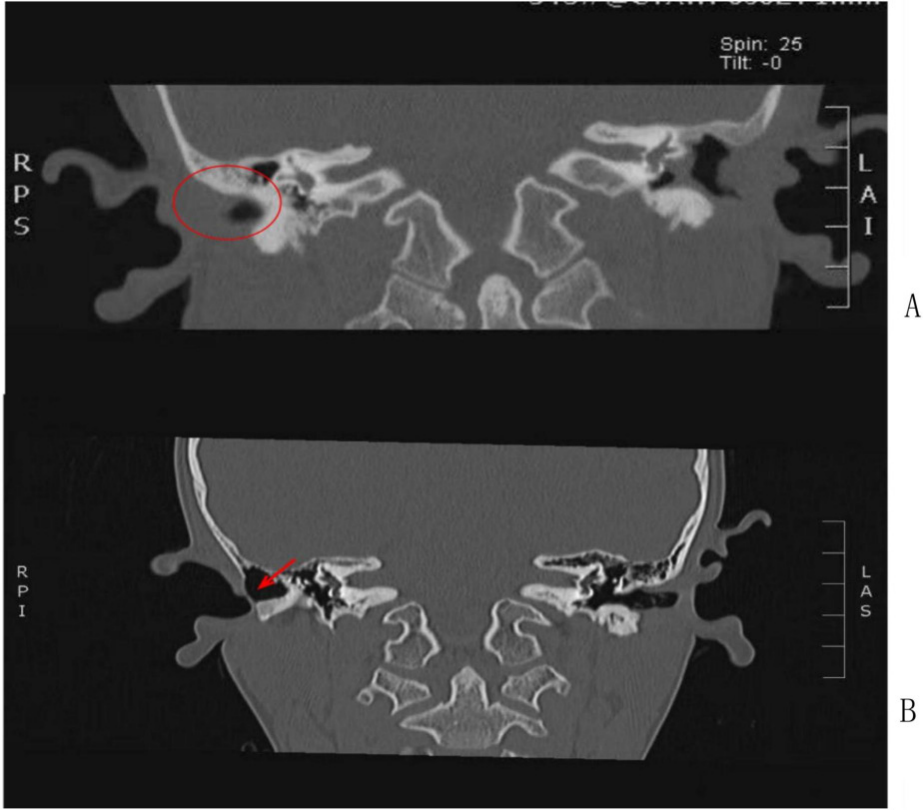
**(A)** temporal bone CT showing complete obliteration of the right EAC lumen by dense fibrous scar tissue, with no visible tympanic membrane. **(B)** The EACH developed 6 months after modified radical mastoidectomy (MRM) for chronic suppurative otitis media and mastoiditis. High-resolution temporal bone CT (axial view) showing linear fibrous obstruction of the right bony EAC with a residual lumen diameter of 1.5 mm, no bony hyperplasia or mastoid air cell involvement.

Diagnosis in children integrates endoscopic evaluation with high-resolution imaging, with strict differentiation from congenital stenosis (all congenital cases were excluded per protocol). We refined the definition of recurrence to encompass new or recurrent otorrhea, tinnitus, or otalgia plus restenosis necessitating reintervention—broadening beyond traditional criteria (e.g., EAC diameter < 3 mm or persistent otorrhea alone) (15, 16). Computed tomography (CT) proved indispensable for grading stenosis severity and guiding surgical planning (17, 18): illustrative findings included linear right EAC obstruction (Case 2; Figure 1) and complete EAC atresia (Case 3; Figure 3), providing objective morphological benchmarks for therapeutic decision-making. This multimodal diagnostic framework enhances outcome assessment fidelity and supports timely, targeted intervention.

Pediatric acquired EACS exhibits a distinct clinical phenotype that differs significantly from its adult counterpart. All pediatric patients presented exclusively with conductive hearing loss; none reported tinnitus, otorrhea, or otalgia. In contrast, among adults, the prevalence of these symptoms was 25% (tinnitus), 50% (otorrhea), and 17% (otalgia). This divergence likely reflects age-specific anatomical determinants: the pediatric EAC is shorter, narrower, and lined with thinner mucosa—features that predispose to immediate conductive impairment upon stenosis onset, without early secondary inflammatory manifestations (14). Furthermore, the postoperative recurrence rate was markedly higher in children (4/7, 57%) than in adults (3/12, 25%; *P* = 0.012), and all pediatric recurrences were histologically and radiologically confirmed re-stenoses. This strongly suggests that re-stenosis is not merely a complication but a defining phenotypic feature of pediatric EACS recurrence—intimately linked to the heightened proliferative and fibrotic capacity of immature tissue (19).

**Figure 2:**
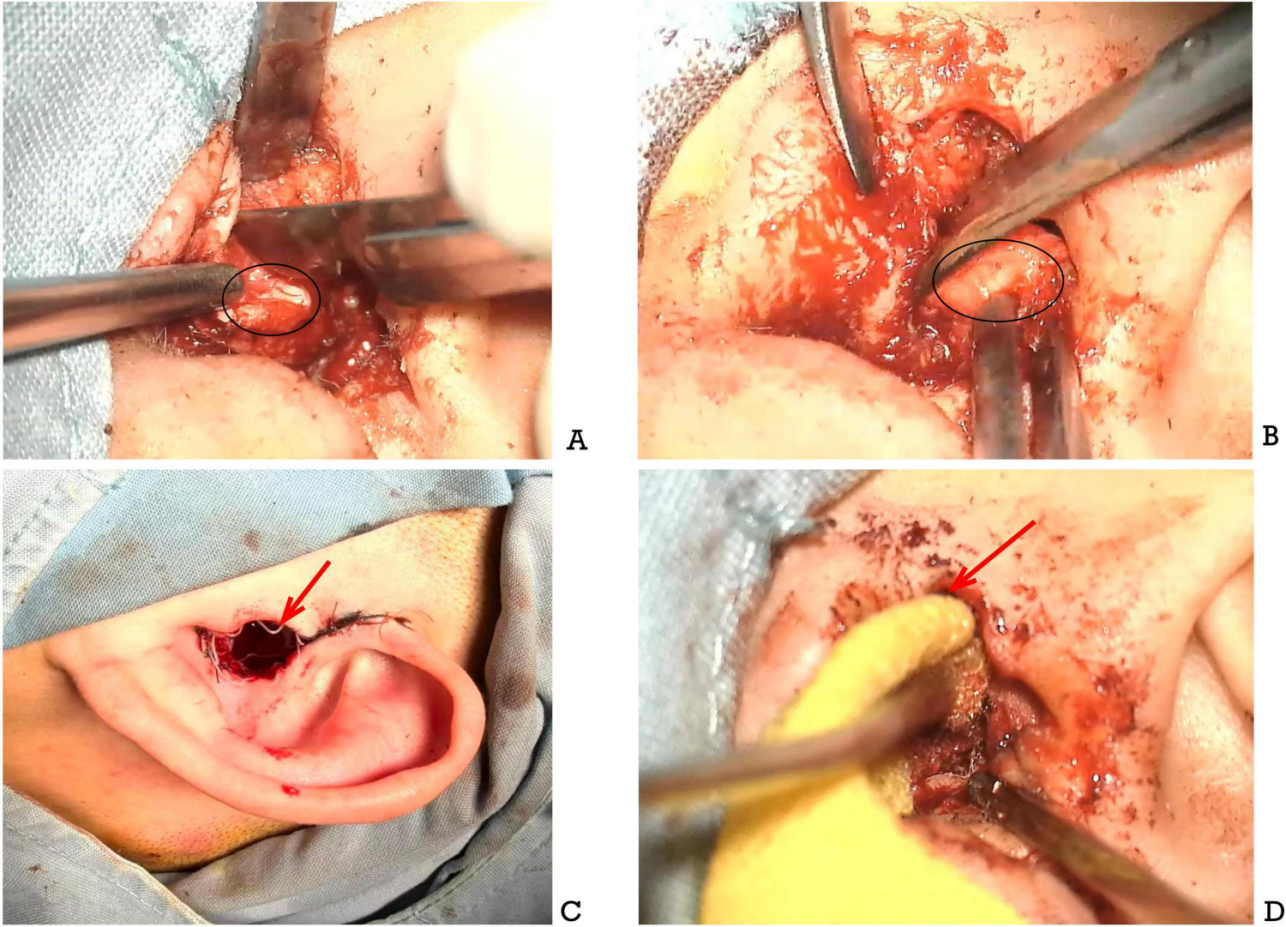
**(A)** and **(B)** show the intraoperative procedures of auricular fossa cartilage resection, which is performed to enlarge the bony external auditory canal; the area marked with a circle indicates the auricular fossa cartilage. **(C)** is the intraoperative image of absorbable drug-eluting stent (DES) implantation after canaloplasty, and the red arrow points to the absorbable drug-eluting stent used in this study [Puyi (Shanghai) Biotechnology Co., Ltd., Model: SDES2514-1]. **(D)** displays the packing procedure with iodoform gauze following stent implantation; the gauze is packed from the tympanic membrane level to the superficial part of the external auditory canal with a conventional length of approximately 10 cm, providing mechanical support for the grafted tissue and preventing postoperative adhesion.

Management of pediatric EACS mandates individualized surgical planning, rigorously tailored to both anatomical constraints and etiological drivers, with dual objectives: restoration of durable EAC patency and mitigation of re-stenosis risk (20, 21). As illustrated in (Figure 2), key procedural steps include targeted cartilage modification, vascularized flap coverage, and adjunctive intracanal support.

Flap selection is critical: the temporalis fascial flap (3/7, 43%) remains the most frequently employed option in children, owing to its robust vascularity, superficial harvest site, versatility in contouring, and technical feasibility—even though it is infrequently utilized in other pediatric otologic procedures (22–24). Emerging evidence supports Thiersch grafts (harvested from the inner thigh or postauricular region) as a viable alternative: their thin, pliable architecture conforms precisely to the narrow pediatric EAC lumen, minimizing volumetric compromise and secondary stenosis risk; high graft take rates enable rapid re-epithelialization of the EAC mucosal barrier, thereby suppressing postoperative inflammation and granulation tissue formation (10, 25). Cartilage resection—including conchal and tragal excision—was performed in 4/7 children (57%), primarily to optimize EAC geometry for long-term luminal stability (Table 4). Notably, neither flap type, cartilage resection, nor drug-eluting stent implantation (6/7, 86% in children vs. 6/12, 55% in adults) demonstrated a statistically significant association with recurrence (*P* > 0.05 for all), a finding likely attributable to limited statistical power given the small cohort size. All procedures were performed via endoscopic ear surgery—a minimally invasive approach uniquely suited to the short, narrow pediatric EAC—though it demands exceptional precision to avoid iatrogenic mucosal trauma.

**Figure 3:**
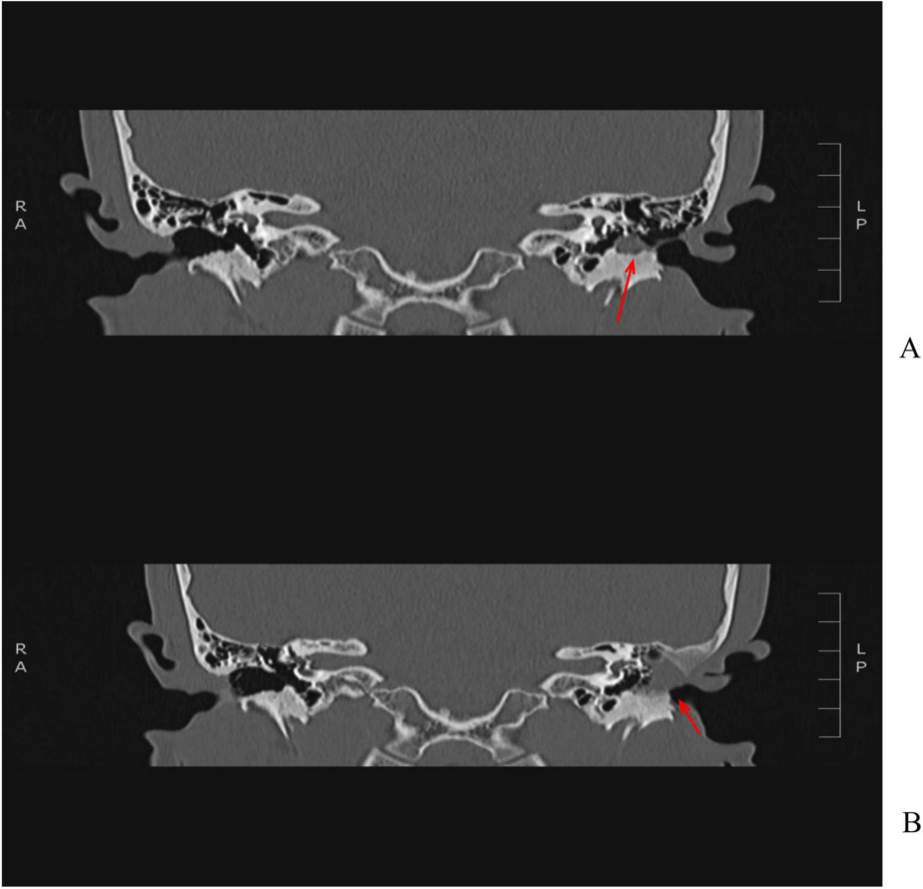
**(A)** (initial admission Ear CT findings) soft tissue density shadows with interspersed bony sclerosis are identified in the left external auditory canal, mastoid air cells, tympanic cavity, and mastoid antrum. The ossicles are embedded and show extensive bony destruction. **(B)** (Follow-up Non-contrast Ear CT Findings 8 Months After Left Mastoidectomy) Partial bony structures of the left mastoid and the entire left ossicular chain are absent. The residual mastoid bone exhibits sclerosis. Soft tissue density shadows are present in the tympanic cavity and mastoid antrum. The left external auditory canal is obliterated.

**Figure 4:**
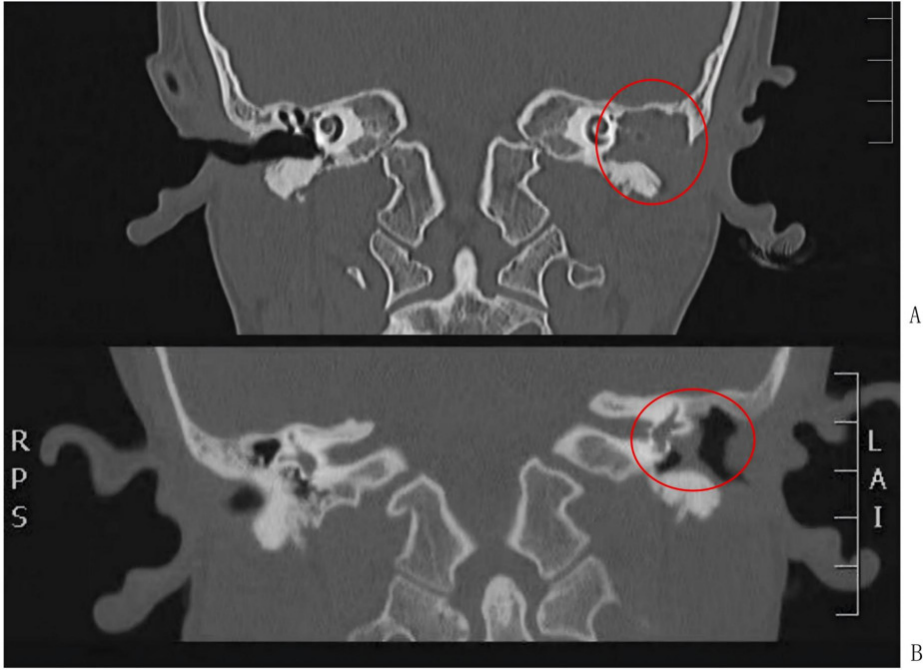
**(A)** soft tissue density shadows are seen in the left external auditory canal, mastoid air cells, tympanic cavity and mastoid antrum, with interspersed osteosclerosis of the bony structures. The auditory ossicles are encased by the soft tissue, and most of the auditory ossicular structures show bony destruction. Bony destruction is also identified in the antral wall with ill-defined anc irregular margins of the destroyed bone, and complete atresia of the external auditory canal is noted. **(B)** Partial bony destruction of the left mastoid and absence of the left auditory ossicular structures are observed, with osteosclerosis of the residual mastoid bone. Soft tissue density shadows are present in the tympanic cavity and mastoid antrum, and obliteration of the left external auditory canal is seen.

Restenosis is the predominant postoperative complication in pediatric acquired EACS, as evidenced by a 57% incidence (4/7) in our cohort. This finding reflects study-specific outcomes and cannot be extrapolated to the broader pediatric EACS population. The high restenosis rate constitutes the principal pathological substrate of postoperative recurrence in children. While intrinsic factors—including anatomical immaturity of the EAC and heightened proliferative/fibrotic potential—contribute significantly to recurrence risk, modifiable clinical variables exert substantial influence. Specifically, treatment delay is associated with progressive fibro-osseous remodeling: in Case 3 (Figure 3), a 5-year interval between symptom onset and surgical intervention preceded recurrent restenosis and radiographically confirmed progressive temporal bone erosion—demonstrating that delayed management exacerbates disease progression and increases procedural morbidity. Concurrently, inconsistent postoperative surveillance impedes early detection of subclinical restenosis and timely therapeutic adaptation; Case 2 (Figure 4) exemplifies this, wherein irregular follow-up directly contributed to diagnostic delay and clinical deterioration. Collectively, these observations underscore that non-adherence to timely intervention and structured longitudinal monitoring compromises therapeutic durability—highlighting the imperative for standardized, age-stratified clinical pathways anchored in prompt referral and protocol-driven surveillance.

Over the past three decades, multiple studies have addressed acquired EACS management (26–28). A consistent limitation across this literature is the confluence of small sample sizes, abbreviated follow-up durations, and procedural heterogeneity in both surgical technique and postoperative protocols—factors that collectively limit the validity and generalizability of long-term outcome assessments. Consequently, rigorous, prospectively scheduled postoperative follow-up is not merely advisable but essential for sustaining durable EAC patency.

Among otologic procedures, modified radical mastoidectomy (MRM) carries the highest attributable risk for iatrogenic EACS—primarily due to extensive soft-tissue dissection, periosteal stripping, and bony cavity creation within the EAC-adjacent anatomy. As illustrated in Case 2 (Figure 1), a 6-month post-MRM CT scan revealed linear lateral EAC obstruction—a direct morphological consequence of unmodulated cicatricial contraction. Given this elevated risk—particularly in children with fragile mucosa and robust healing responses—prophylactic strategies must be deliberately integrated into the surgical workflow. We therefore propose a four-pillar prevention framework:

1. Perioperative implantation of absorbable, steroid-eluting drug-eluting stents (DES), standardized in composition, dosing, and retention duration (3–6 months), to concurrently suppress fibroblast hyperactivity and provide mechanical luminal scaffolding (29);
2. Prioritization of endoscopic, minimally invasive approaches to minimize iatrogenic trauma to EAC epithelium and adjacent perichondrium—preserving native tissue integrity as the primary anti-fibrotic strategy;
3. Protocolized postoperative topical therapy: scheduled replacement of antibiotic-steroid–impregnated packing, coupled with adjunctive corticosteroid irrigation, to mitigate inflammation and optimize mucosal re-epithelialization;
4. Mandatory longitudinal surveillance: monthly clinical-endoscopic assessment for the first 6 months postoperatively, enabling early detection of asymptomatic restenosis, timely debridement, and staged DES exchange.

Postoperative packing serves a critical mechanobiological role in mitigating restenosis following surgical intervention for acquired EACS. Packing is routinely initiated immediately after hemostasis and removed at 2–4 weeks postoperatively to prevent biofilm-associated infection secondary to retained exudate-debris complexes (13). Evidence supports the efficacy of topical steroid-antibiotic combinations in suppressing postoperative inflammation and serous secretion. To optimize localized drug delivery—particularly within the anatomically constrained stenotic EAC—we recommend pre-saturation of iodoform gauze with compounded steroid-antibiotic solutions, combined with concurrent placement of absorbable, steroid-eluting drug-eluting stents (DES) (13, 29–32). As complete EAC re-epithelialization requires ≥30 days (33), monthly clinical-endoscopic surveillance is essential to guide timely packing removal, wound bed debridement, and staged DES exchange—thereby preventing epithelial gap formation and secondary luminal collapse. Hydrophilic expandable ear cores—composed of hydroxylated polyvinyl acetate—represent an emerging alternative: upon *in situ* hydration, they generate gentle, circumferential radial pressure that maintains patency while minimizing mucosal shear stress relative to conventional gauze (13, 34). This biomechanical advantage is especially valuable in pediatric patients, whose immature EAC epithelium exhibits heightened susceptibility to friction-induced injury.

Several limitations of this study warrant explicit acknowledgment. First, the retrospective, single-center design—enrolling only 7 pediatric and 12 adult patients—imposes inherent constraints on statistical power and external validity. With only four pediatric recurrences (4/7), correlation analyses were severely underpowered, increasing susceptibility to Type II error (i.e., false-negative associations). Second, postoperative follow-up was incomplete in multiple cases due to loss to follow-up, compromising longitudinal assessment of restenosis incidence, hearing outcomes, and prognostic factor identification across both age groups. Critically, the median follow-up duration (≤24 months) is insufficient to capture the full developmental trajectory of the pediatric EAC or evaluate the durability of absorbable stent interventions beyond the initial fibroproliferative phase. Third, subgroup analyses—including stratification by surgical timing, stent composition, or etiological subtypes—were not performed owing to sample size limitations. Future multicenter prospective studies with larger cohorts and extended follow-up (>36 months) are essential to validate optimal perioperative protocols and elucidate the molecular and cellular mechanisms underlying pediatric EACS restenosis.

## 5 Conclusion

To date, the literature on acquired EACS remains predominantly adult-centric, with a notable paucity of data characterizing the distinct clinical phenotype, optimal surgical strategy, and evidence-informed postoperative management protocols for pediatric patients. Addressing this gap, the present study undertook a comparative cohort analysis of pediatric and adult EACS, systematically delineating differences in demographic profiles, etiologic patterns, operative techniques, and outcomes—with particular focus on how age-specific postoperative regimens influence restenosis risk. While the observed higher restenosis rate in children and its consistent association with younger age support age as a clinically relevant prognostic determinant, the retrospective, single-center nature of this investigation precludes definitive causal inference. Therefore, prospective, multicenter validation is required to establish age as an independent predictor of restenosis. Furthermore, given the histopathological hallmark of exaggerated fibroproliferative activity in pediatric EACS, future mechanistic studies should prioritize the identification and functional validation of dysregulated molecular effectors as foundational steps toward developing targeted adjuvant antifibrotic therapies.

## Data Availability

The datasets presented in this study can be found in online repositories. The names of the repository/repositories and accession number(s) can be found in the article/Supplementary Material.

## Supplementary material

Supplementary Table S1 Postoperative follow-up data of patients with EACS.
