# Supplementary material for: Acquired external auditory canal stenosis: clinical characteristics, surgical strategies and prognostic analysis
Source: Front Pediatr. 2026 May 14;14:1814290. doi: 10.3389/fped.2026.1814290 (PMC13216209; doi:10.3389/fped.2026.1814290)
Supplement: Supplementary Table S1 — Postoperative follow-up data of patients with EACS. [file Table1.docx]

**Table S1** Postoperative Follow-up Data of Patients with EACS

| Group | Case No. | Age (y) | Sex | Specific Surgical Cause | Time interval between Primary Surgery and Stenosis/Atresia Onset | Postoperative Restenosis |
| --- | --- | --- | --- | --- | --- | --- |
| Pediatric (n=6) | 1 | 8 | F | MRM | 8months | N |
|  | 2 | 14 | F | MRM | 6months | Y |
|  | 3 | 8 | M | MRM | 10years | Y |
|  | 4 | 14 | M | MRM | 5years | Y |
|  | 5 | 8 | M | Canaloplasty | 4years | Y |
|  | 6 | 8 | M | Excision of a mass behind the left ear | 12years | N |
| Adult（n=6） | 4 | 53 | F | MRM | 1years | N |
|  | 5 | 51 | F | MRM | 0.5month | N |
|  | 8 | 41 | F | Excision of a mass behind the left ear | 2years | N |
|  | 9 | 31 | M | MRM | 12years | N |
|  | 11 | 46 | F | MRM | 2years | N |
|  | 12 | 60 | F | MRM | 12years | N |

This table includes only patients with EACS related to prior ear procedures (6 paediatric and 6 adult cases); those with traumatic/post-inflammatory aetiologies are excluded.

Abbreviations：F=female；M=male，R=right；L=left；B=bilateral，Y=Yes;N=No；y=years；Age refers to the age at the time of the initial surgery.

Time Interval: The period from the initial ear surgery to the onset and confirmed diagnosis of external auditory canal stenosis/occlusion.MRM ：Modified Radical Mastoidectomy
